# Maladaptive alterations of resting state cortical network in Tinnitus: A directed functional connectivity analysis of a larger MEG data set

**DOI:** 10.1038/s41598-019-51747-z

**Published:** 2019-10-29

**Authors:** Evangelos Paraskevopoulos, Christian Dobel, Andreas Wollbrink, Vasiliki Salvari, Panagiotis D. Bamidis, Christo Pantev

**Affiliations:** 1 School of Medicine, Faculty of Health Sciences, Aristotle University of Thessaloniki, PO Box 376, P.C. 54124 Thessaloniki, Greece; 2 Institute for Biomagnetism and Biosignalanalysis, University of Münster, Malmedyweg 15, P.C. D-48149 Münster, Germany; 3 Department of Otorhinolaryngology, University Hospital Jena, 07740 Jena, Germany

**Keywords:** Cortex, Perception

## Abstract

The present study used resting state MEG whole-head recordings to identify how chronic tonal tinnitus relates to altered functional connectivity of brain’s intrinsic cortical networks. Resting state MEG activity of 40 chronic tinnitus patients and 40 matched human controls was compared identifying significant alterations in intrinsic networks of the tinnitus population. Directed functional connectivity of the resting brain, at a whole cortex level, was estimated by means of a statistical comparison of the estimated phase Transfer Entropy (pTE) between the time-series of cortical activations, as reconstructed by LORETA. As pTE identifies the direction of the information flow, a detailed analysis of the connectivity differences between tinnitus patients and controls was possible. Results indicate that the group of tinnitus patients show increased connectivity from right dorsal prefrontal to right medial temporal areas. Our results go beyond previous findings by indicating that the role of the left para-hippocampal area is dictated by a modulation from dmPFC; a region that is part of the dorsal attention network (DAN), as well as implicated in the regulation of emotional processing. Additionally, this whole cortex analysis showed a crucial role of the left inferior parietal cortex, which modulated the activity of the right superior temporal gyrus, providing new hypotheses for the role of this area within the context of current tinnitus models. Overall, these maladaptive alterations of the structure of intrinsic cortical networks show a decrease in efficiency and small worldness of the resting state network of tinnitus patients, which is correlated to tinnitus distress.

## Introduction

Chronic tinnitus is the experience of a persistent auditory phantom percept, being heard in the absence of an external source for a period of time longer than three months^1^. It affects 9–15% of the general population, while a subtle, transient tinnitus percept has a prevalence of more than 30%^2,3^. The continuity of tinnitus percept is related to significant distress and reduction in quality of life, accompanied by sleeping problems, stress, depression and substance abuse^4^. Several models have been proposed in order to explain the factors that generate tinnitus^5–8^, highlighting its association with maladaptive subcortical and cortical processes such as hyperactivity, burst firing and hyper-synchronicity, following hearing loss^9^. Nonetheless, our understanding of its underlying physiological mechanisms remains insufficient, while recent evidence suggests that the percept of tinnitus is linked with altered interaction between intrinsic cortical networks^10–12^.

The initial generation of this phantom, but extremely bothersome percept is thought to be caused by damage to the auditory periphery, leading to functional de-afferentation of the tonotopically arranged auditory neurons^13^. The de-afferented neurons start to respond preferentially to frequencies neighboring the hearing loss region, resulting in reduced lateral inhibition onto neurons coding the audiometric edge. Maladaptive plasticity processes follow, reorganizing the tonotopic structure to compensate the lack of lateral inhibition^8,14^. This working model of tinnitus, partially confirmed in animal studies^15^, does not fully explain its chronification, nor the discomfort caused by tinnitus. Instead, findings related to the contribution of emotional and attentional processes^12,16^, involving wide spread cortical networks, may account for its conversion from transient to chronic tinnitus via a predictive mechanism^7^.

Several recent neuroimaging studies explored the altered activation and/or brain connectivity of tinnitus patients in the resting state. These studies highlight the tinnitus-related reorganization of connectivity between intrinsic auditory, limbic, and attention networks as well as changes in the way that the Default Mode Network interacts with other resting state networks^12,17,18^. Brain regions consistently found to exhibit altered connectivity in tinnitus patients, compared to controls, include the auditory cortices and its connectivity to middle temporal and para-hippocampal sources^18^; the precuneus and its connectivity to the dorsal prefrontal cortex and the dorsal attention network^17,19^. Nonetheless, these studies also revealed some controversial findings, as alterations in the connectivity of primary auditory cortices are not consistently found^20^.

These inconsistencies may be caused by the relatively small sample size of tinnitus patients commonly used, which may not incorporate the heterogeneity of the population. Moreover, characteristics of the tinnitus percept such as loudness, laterality, duration^21^ and efficiency of coping^22^ may play an important role in how tinnitus affects resting state connectivity^16^. Additionally, fMRI studies investigating resting state connectivity in tinnitus patients exhibit a high spatial resolution, but three inherent limitations: (a) low temporal resolution, (b) most of the studies use predefined seeds for the analysis of the connectivity^23^, limiting the possibility to find direct connections between sources which are not included in the corresponding seed regions; and (c) the scanner noise, which is impossible to be completely eliminated^24^. This strong acoustic signal interferes with resting state of the auditory processing pathway^25^, an interference which may influence tinnitus patients to a different extent than controls, e.g. by masking tinnitus and therefore interfering with the phenomenon itself^10^. On the other hand significant differences between tinnitus patients and controls in connectivity have been also documented by means of EEG^26^, highlighting the role of stress in the perception of tinnitus and the role of parahippocampus as a node of a network, which includes additionally the posterior cingulate cortex, the anterior cingulate, the insula and the auditory cortices. The MEG study by Schlee *et al*.^22^, concluded that the temporal cortices show hyperactivity in tinnitus patients and integrate to a global network of long-range cortical connectivity.

According to the best of our knowledge, the majority of the previous studies as outlined above, investigated resting state networks via functional connectivity and, therefore, were not able to indicate directionality of the connectivity differences; while a minority of the corresponding studies used directed functional connectivity metrics^18,22,27^.

Therefore, the goal of the present study was to investigate directed functional connectivity of intrinsic cortical networks underpinning tinnitus via resting state whole-head magnetoencephalographic measurements. Via this approach we aimed to quantify tinnitus-related alterations in directed functional connectivity of cortical regions related to the extensive discomfort caused by tinnitus. To this aim we used MEG to record resting state cortical activity of a group of tinnitus patients and a matching group of control subjects. Source analysis was performed using low resolution electromagnetic tomography (LORETA)^28^ in order to solve the inverse problem, whereas directed functional connectivity between all combinations of the corresponding source time series was estimated via phase transfer entropy (pTE)^29^ in the frequency range of 0.5–35 Hz and the application of a statistical model to extract significant connections of the network. Importantly, this method allows to determine the direction of flow and therefore to describe regions with a modulating role on others. Hence, a whole head, node-to-node network analysis was performed, using a grid of 863 nodes, without *a-priori* defined model of regions of interest, following a graph-theoretical approach. We hypothesized that tinnitus patients will show increased connectivity between auditory, medial temporal and para-hippocampal sources as well as between regions of the medial temporal cortex and sources of the dorsal attention network and the frontal lobe. Lastly, as the resting state network of healthy adults has been found to exhibit a global structure showing computational efficiency and small-worldness^30^, and maladaptive neuroplastic processes seem to alter these networks^31^, we hypothesized that the tinnitus-related changes in directed functional connectivity will significantly affect these global characteristics of intrinsic cortical networks of tinnitus patients by reducing network efficiency and small-worldness.

## Results

### Cortical network analysis results

**Figure 1:**
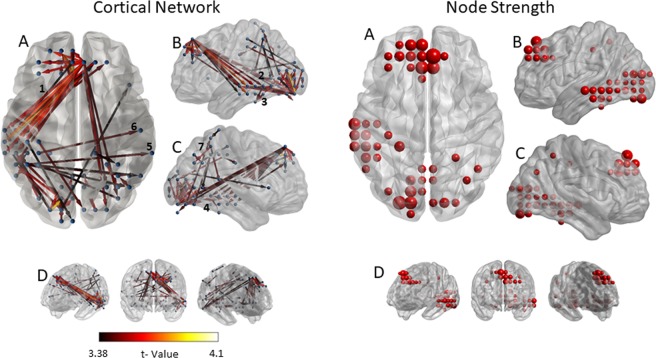
Differences in cortical connectivity between Tinnitus Patients and Controls. Left side: Statistical map of brain regions (contrast: patients > controls) showing significantly greater connectivity in the group of tinnitus patients in comparison to controls. Connectivity is depicted via line arrows between brain regions. Networks presented are significant at p < 0.001, FDR corrected. The color scale indicates t-values. Significant differences between tinnitus patients and controls are depicted via direct connections (1) from the dorsal prefrontal cortex to left medial temporal and para-hippocampal sources, (2) from lateral occipital to left medial temporal and para-hippocampal sources, (3) from the left medial temporal to the left inferior temporal gyrus, (4) from the right fusiform to the right dorsal prefrontal cortex, (5) from the left inferior parietal cortex to the posterior part of the right superior temporal gyrus, (6) from the left postcentral gyrus to the right medial temporal gyrus and (7) a local connectivity pattern between precuneus and posterior parietal sources. Figure shows (A) Top view, (B) Lateral view, left (C) lateral view, right, (D) frontal view −45°, frontal view, frontal view +45°. Right side: Node strength of the significant networks. Node strength sums the weights of links connected to the node, identifying the brain regions that show greater connectivity differences. Strength is represented by node size. Figure shows (A) Top view, (B) Lateral view, left (C) lateral view, right, (D) frontal view −45°, frontal view, frontal view +45°. Results indicate a cluster of nodes (with increased node strength and hence increased role within the network) in the dorsal prefrontal cortex, the left medial temporal and parahippocampal regions, and the left inferior temporal and lateral occipital gyrus.

The statistical comparison of the adjacency matrices of the two groups revealed that the resting state activity of the tinnitus patients had significantly stronger connectivity between several cortical sources [P < 0.001; false discovery rate (FDR) corrected] comprising a network with 40 nodes and 60 edges. As pTE generates a non-symmetric adjacency matrix, the network differences identified allow directional connectivity inferences to emerge from the data. Therefore, information regarding the direction of the connectivity is included in the description that follows. This network consisted of direct connections from the dorsal prefrontal cortex to left medial temporal and para-hippocampal sources and from the left inferior parietal cortex to the posterior part of the right superior temporal gyrus, the bilateral middle temporal and left visual association areas. In addition, significantly greater connectivity in the group of tinnitus patients, compared to controls, was identified as a direct connection from lateral occipital to left medial temporal and para-hippocampal sources, from left medial temporal sources to left inferior occipital ones, from the right fusiform to the right dorsal prefrontal cortex and a local connectivity pattern between precuneus and posterior parietal sources. The node strength analysis sums the weights of links connected to each node, identifying the brain regions that show greater connectivity differences. This analysis indicated that the nodes with the greater sum of connectivity weights of this network were located in the dorsal prefrontal cortex, in the left medial temporal and the lateral occipital gyrus. The statistical map of this analysis, along with the corresponding node strengths are presented in Fig. 1. The opposite contrast, evaluating connectivity with greater strength in the group of controls, compared to the group of patients, yielded no significant results. Alongside, the application of the exact same analysis pipeline to a set of appropriate surrogate data, yielded no significant result, even when the threshold of statistical significance was set to p < 0.1 (corrected for multiple comparison via FDR correction). This fact indicates that the obtained group differences cannot be attributed to biases in the estimation of directional connectivity, but rather due to significant differences across the two groups (i.e. tinnitus patients and controls).

The statistical comparison of graph characteristics revealed a significant decrease in small worldness propensity [mean SWPpatients = 0.956; mean SWPcontrols = 0.978; U = 2.207; p = 0.027], a significant decrease in efficiency [mean Epatients = 1.093; mean Econtrols = 1.115; U = 2.159; p = 0.031] and a significant increase in characteristic path length [mean CPLpatients = 0.9223; mean CPLcontrols = 0.902; U = −2.275; p = 0.023]. Density of the significant networks for each group was estimated at Dpatients = 0.0024 and Dcontrols = 0.0012. Density was measured for the significant, group level networks and therefore, no variance across the subjects was available.

**Figure 2:**
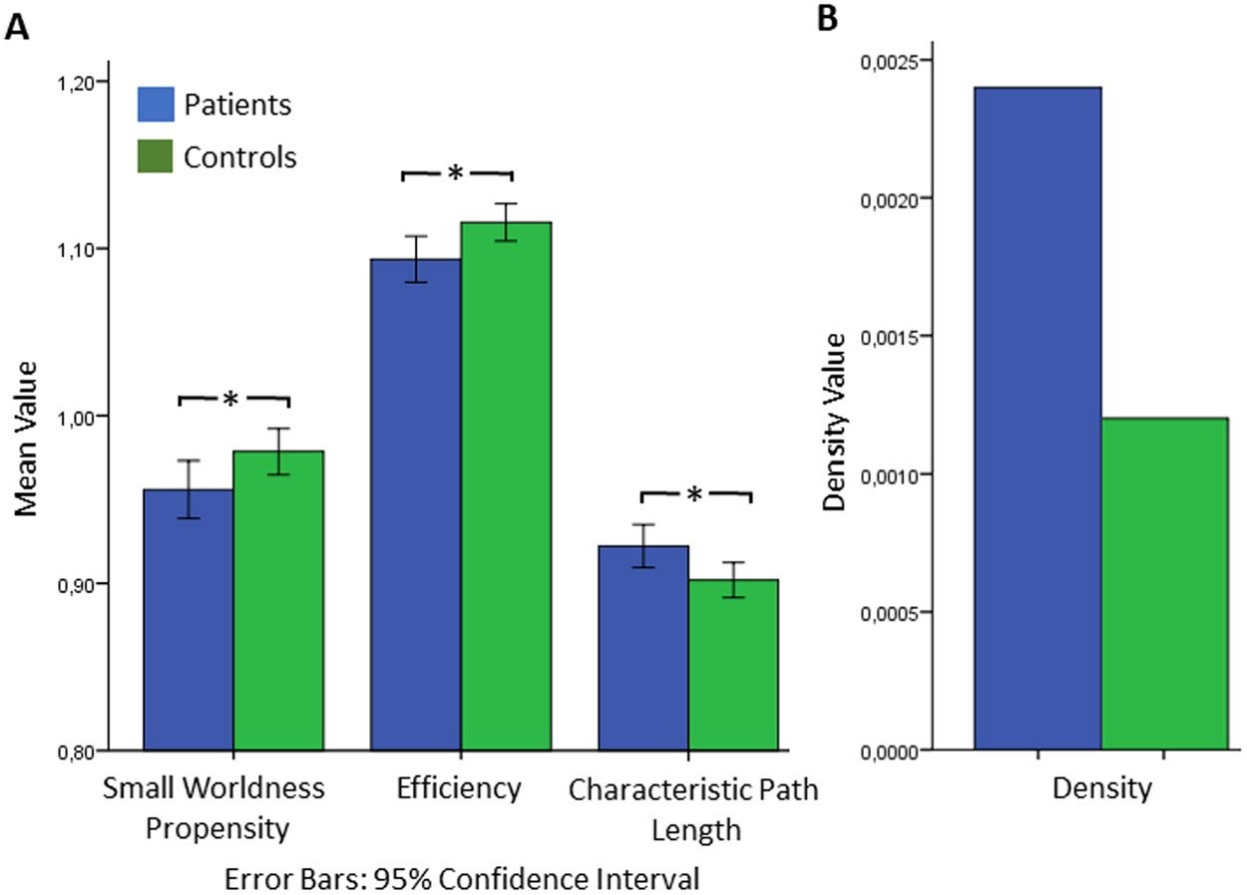
Global characteristics of the resting state network of tinnitus patients (blue color bar) and healthy controls (green color bar). Bar plot A includes the graph measures Small Worldness Propensity, Characteristic Path length, and Efficiency. *indicates that non-parametric Mann-Whitney U value of the comparison between the two groups is significant at p < 0.05. Bar plot B includes Density. Density was measured for the, significant, group level networks and therefore, no variance across the subjects was available. Hence, this measure is presented as a qualitative description of the statistical result derived from the connectivity analysis and not as a quantitative statistic.

The resting state network of the group of tinnitus patients was revealed by the statistical analysis of their non-symmetric pTE adjacency matrices, indicating a complex system of sources. The graph [P < 0.001; false discovery rate (FDR) corrected], compiled by 234 nodes and 892 edges, depicted connectivity patterns between angular and anterior sources as well as between medial temporal and frontal sources. As seen in Fig. 4, this network included also sources which are linked with resting state networks such as the DMN, the auditory, the visual and the lateral fronto-parietal network. The global qualitative characteristics of the graph (i.e. small worldness propensity, characteristic path length, efficiency and density) are presented in Fig. 2.

Tinnitus patients scored in Tinnitus Handicap Inventory (THI) a mean of 23.81 (SD = 11.07), in Visual Analog Scale (VAS) measuring perceived tinnitus loudness a mean of 42.33 (SD = 25.93), in the VAS of distress a mean of 23.81 (SD = 11.07) and in the VAS of handicap a mean of 31.47 (SD = 24.21). The correlation analysis between the graph measures of small worldness propensity, characteristic path length and efficiency with loudness, distress, THI and hearing loss, showed that tinnitus loudness and hearing loss did not correlate with the network characteristics. Nonetheless, tinnitus distress did show several correlations: specifically, tinnitus distress, as measured by the VAS showed significant positive correlation with characteristic path length [r = 0.571; p = 0.011] and significant negative correlations with small worldness propensity [r = −0.650; p = 0.003] and efficiency [r = −0.602; p = 0.006]. In addition, THI showed a significant negative correlation with efficiency [r = −0.658; p = 0.001], a small but significant negative correlation with small worldness propensity [r = −0.465; p = 0.029] and a positive correlation with characteristic path length [r = 0.613; p = 0.002].

**Figure 3:**
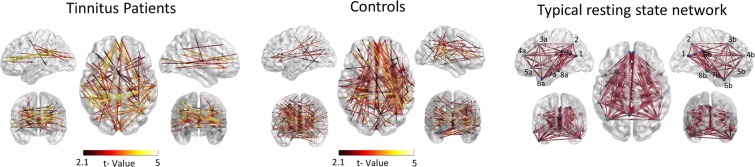
Resting state cortical connectivity network for each group. Left side: Connectivity of the whole-cortex resting state network of tinnitus patients. Middle side: Connectivity of the whole-cortex resting state network of controls. Similarly to tinnitus patients, the resting state network of healthy controls shows significant connectivity between all brain regions typically correlated with brain’s resting state. Right side: Full graph (fully connected network) of a typical whole-cortex resting state network as derived from the literature^69,70^. It should be clarified here, that many canonical resting state networks exist^71^, and the one presented here depicts the default mode network. Regions included in the typical resting state network are the following: (1) precuneus, (2) posterior cingulate, (3a) middle frontal gyrus left, (3b) middle frontal gyrus right, (4a) medial frontal gyrus left, (4b) medial frontal gyrus right, (5a) anterior cingulate left, (5b) anterior cingulate right, (6a) inferior temporal gyrus left, (6b) inferior temporal gyrus right, (7a) parahippocampal gyrus left, (7b) parahippocampal gyrus right, (8a) middle temporal gyrus left, (8b) middle temporal gyrus right, (9a) inferior parietal lobule left, (9b) inferior parietal lobule right.

The resting state network of the group of healthy controls was revealed by the statistical analysis of the corresponding connectivity matrices and, similarly to the analysis of the patients, depicted a complex system of cortical regions. The graph [P < 0.001; false discovery rate (FDR) corrected], included 425 nodes, and 441 edges. The network showed greater connectivity strength between sources of the right hemisphere and a morphology that, due to the increased number of sources participating in the network, includes all resting state networks, such as the auditory, the sensory-motor, the visual and the lateral fronto-parietal network (Fig. 3).

## Discussion

The present study used resting state MEG recordings to identify how chronic tonal tinnitus relates to altered functional connectivity of intrinsic cortical networks. The resting state MEG activity of 40 chronic tinnitus patients and 40 controls was compared identifying significant alterations in intrinsic networks of the tinnitus population. Directed functional connectivity of the resting brain, at a whole cortex level, was estimated by means of a statistical comparison of the estimated phase Transfer Entropy (pTE) between the time-series of cortical activations, as reconstructed by LORETA. As pTE, identifies the direction of the information flow, a detailed analysis of the connectivity differences between tinnitus patients and controls was possible. Results indicated that the group of tinnitus patients showed increased connectivity between dorsal prefrontal and right medial temporal areas, with the former mostly affecting the later. The resting state networks of both groups showed small-worldness, nonetheless, the group of tinnitus patients had significantly reduced small worldness in comparison to controls, a characteristic which was accompanied by significantly decreased network efficiency and increased characteristic path length. These results indicate that chronic tonal tinnitus is related to altered functional connectivity of intrinsic cortical networks, showing increased engagement of the affective and attentional networks in the resting state of tinnitus patients, along with an effect in the auditory domain.

Connectivity of distant and functionally distinct cortical areas, which form distributed networks of synchronized brain activity, is essential for information processing^32^. MEG, measuring the physiological activity of the cortex in a direct way and with high temporal resolution, constitutes one of the most attractive techniques for identifying directed functional brain connectivity^33^. The default mode network, as measured by fMRI studies, is reflected in alpha and beta band of MEG data^34^, while the resting activity measured with MEG provides additional information regarding the connectivity of other intrinsic networks, such as the dorsal attention and affective network, which are reflected in different electrophysiological bands when at rest^33,35^. Hence, the analysis of a wide frequency spectrum of MEG data (i.e. 0.5–35 Hz) allowed us to investigate linear and non-linear tinnitus driven interactions between different resting state networks, while at the same time estimate the directionality of the connectivity. Such interactions may be grounded on the modulation of the power of higher frequency bands by the phase of lower frequency signals^36^. Higher frequencies (35+ Hz) where excluded as the analysis focused on cortical activity, as measured by MEG.

The most prominent result of this whole head analysis indicates that tinnitus patients exhibit a stronger connectivity, in comparison to healthy controls, between the dorsomedial prefrontal cortex (dmPFC) and left medial temporal sources, such as the para-hippocampal gyrus and the middle temporal gyrus. This result is corroborated by the node strength analysis, which identified the dmPFC as the node with the greater sum of connectivity weights. The direction of the information processing pathway as reflected in pTE indicates that dmPFC modulates the activity of left medial temporal sources. This result is in line with a number of recent fMRI resting state studies, which indicate that tinnitus patients show increased activity in the para-hippocampal gyrus^10^ as well as increased connectivity of this area to the auditory cortices and the DMN^12,19^. As para-hippocampal gyrus is an essential node of the affective network, an enhanced involvement of this network in the resting state of tinnitus patients has been shown^37^, and this may be interpreted as a reflection of the emotional distress that these patients exhibit. Our results go beyond previous findings by indicating that the role of this area is dictated by a modulation from dmPFC; a region that is part of the dorsal attention network (DAN), as well as implicated to the regulation of emotional processing^38^. The causal relationship of dorsal prefrontal regions to the tinnitus percept has been also shown by top-down modulation of tinnitus intensity via tDCS applied on dorsolateral prefrontal cortex^39^ and is linked with the maintenance of chronic tinnitus^17,40^.

The whole cortex analysis also showed an increased role of the left inferior parietal cortex, which modulated the activity of the right superior temporal gyrus, bilateral middle temporal and left visual association areas. This result indicates that the DAN may be more directly involved in tinnitus perception, than by a secondary modulation of its activity by precuneus, as Schmidt *et al*.^17^, showed. Instead, this finding is more in line with the model proposed by De Ridder *et al*.^6^, who suggests that the inferior parietal cortex is a core region in the tinnitus network. De Ridder’s model argues that inferior parietal areas are linked to auditory memory and awareness^41^ and due to this functionality this area may account for appointing this phantom percept to an external source. Transcranial magnetic stimulation in this region may also modulate tinnitus^41^, indicating a causal role of this area to the tinnitus percept.

The left inferior parietal area moreover modulates the role of visual association areas, typically involved in the visual resting state network^42^. Increased functional connectivity between auditory and visual resting state networks, which include occipital and temporal-occipital regions, has previously been documented^12,43^, and the authors interpreted this result as a decrease in the spontaneous activity of the visual cortex that is irrelevant to the processing the phantom sound. The directionality of our current analysis allows us to propose that the decrease of the activity of the visual resting state network in tinnitus is dictated by the DAN. The visual network in turn, having a strong role in the overall functionality of the network, as indicated by the node strength analysis, sends information in left medial temporal areas, including the para-hippocampal gyrus, probably serving as a feedback loop in the network^43^.

Our results are also partly in line with the recent study of Mohan *et al*.^27^. The results of this, whole head, directed functional connectivity study indicate a set of cortical areas which play a significant role in the tinnitus distress network, that is highly similar to the regions identified by our study (i.e. left medial temporal areas such as the hippocampal gyrus and the parahippocampal area, the left inferior temporal gyrus and the prefrontal regions of anterior cingulate cortex and the right ventrolateral prefrontal cortex). Nonetheless, in contrast to our results the study of Mohan *et al*.^27^ shows a decrease in the connectivity of these regions, despite the opposite a priori hypothesis of the authors. This outcome is interpreted by the authors as a result of the use of directed functional connectivity metric in contrast to previous undirected analyses. However, as our study also used a directed functional connectivity metric which indicated increased connectivity, the hypo-connectivity found in the Mohan *et al*.^27^ study may be interpreted by the fact that they used a different estimator of directed connectivity incorporating diverse parameters of predicted interaction (such as time delay) or by the fact that only 19 electroencephalographic channels were used in that study to reconstruct the cortical activity, a fact which affects the stability and source separability of the inverse method applied^44^.

Interestingly, the contrast evaluating whether tinnitus patients exhibit significantly decreased connectivity between cortical regions, showed no significant result. This result comes in contrast to previous fMRI studies investigating differences in functional connectivity between tinnitus patients and controls^43,45^, which revealed decreases in functional connectivity between the auditory cortex and the visual cortex^43^ or the prefrontal cortex^45^. A decrease in functional connectivity was not found, though, in the EEG study of^26^, and our interpretation of this finding relies on the difference of the characteristics of the methodology and the neuroimaging modality used (fMRI vs. EEG or MEG). This finding is also in line with the involvement of hyperactivity, burst firing and hyper-synchronicity in tinnitus generation^46^, as these brain mechanisms may support an increase of directed functional connectivity.

In terms of the global qualitative characteristics of the resting state activity, tinnitus seems to alter basic graph features, but without disrupting the network. Hence, the resting state network of tinnitus patients retains small world structure, as would be expected by a biological network that supports efficient parallel information transfer at relatively low cost^47^, but tinnitus reduces significantly its small worldness value, indicating a network with decreased efficiency in transmission of information^48^. In addition, the characteristic path length of the network is significantly increased, showing that the average shortest path length in the network is greater in the tinnitus patients, and hence, information processing is less efficiently integrated between segregated parts of the network^49,50^. Overall, this alteration of network characteristics results in a significant decrease of global efficiency, indicating that the intrinsic network of tinnitus patients has reduced efficiency in comparison to controls^43^.

Global efficiency measures the distance (i.e., number of edges) between any pair of nodes in a network^51^, quantifying the extent of overall communication efficiency of a network. A greater distance means lower routing efficiency because information exchange needs more steps to be completed; therefore the estimation of global efficiency is primarily influenced by short paths^49^. In highly modular networks, with multiple independent components global efficiency is decreased as the path length between disconnected nodes is infinite, and hence efficiency is zero. Hence, global efficiency can be identified as a measure which quantifies, concurrently, a) energy consumption in information processing and b) degree of modularity. Therefore theoretically, if all other characteristics remain stable between two networks which aim to perform similar functionalities, a network showing increased connectivity (i.e. involving more nodes) will exhibit decreased efficiency. Consequently, our results strongly indicate that tinnitus-related reorganization of intrinsic cortical networks reduces the system’s processing economy and induces a more modular network structure. This interpretation is supported by the additional finding that tinnitus distress and the THI show a significant negative correlation with efficiency, indicating that the higher the distress, the smaller the efficiency of the intrinsic brain network and therefore, the smaller the system’s processing economy.

The results of the present study may be translated at a therapeutic approach by the development of intervention protocols which will aim to reduce the modular structure of resting state network of tinnitus patients. Employing our cortical connectivity results, this protocol should decrease the active role of dmPFC in modulating medial temporal areas. Such an approach may be grounded on stimulation techniques such as TDCS or TMS, expanding recent results^39,41^.

The current study did not use a head model based on individual MRIs for the inverse solution of the MEG activity, and this may be interpreted as a limitation since individual head models improve the localization accuracy of MEG^52^. The basic reason, for which we decided not to acquire individual MRIs, was that we did not want to expose our patients to the MRI noise, which very often causes discomfort to tinnitus patients. Instead, an average Finite Element Model based on MNI brain was used, as provided by BESA. Another limitation is the relative heterogeneity of our patients, evidenced by the variance of tinnitus duration, which may affect the corresponding cortical processes^21^ and by the fact that some of the patients experienced a lateralized tinnitus percept. The effect of both of these issues, however, should be limited by the increased sample size that our study incorporated, in comparison to previous studies. With regard to the parameters for the calculation of PTE one has to note that use of a delay on both source and target, as well as the number of bins may lead to inflated PTE values. Specifically, the number of bins used results in undersampling, as it means that 10 data samples are averaged per bin for each marginal entropy. Nonetheless, these settings apply to both groups (patients and controls) as well as the surrogate data used for the validation of our analysis, and hence should not affect the group statistics, nor the conclusion reported. Alongside, the use of PTE for estimating cortical connectivity has the advantage of taking into account non-linear relationships between different resting state networks, as it does not disentangle the different frequency bands. Therefore, PTE is a very suitable metric to evaluate broadband connectivity. Nonetheless, this results in an inability to apply the classical interpretation of feedforward and feedback information processing which may be expressed in different frequency bands^53^ and has been found to provide a meaningful interpretation of tinnitus related neurophysiology^54^. This limitation is partly overcome by the directionality of the connectivity result, as this information, instead, may lead to feedforward and feedback information processing interpretations of the connections. Finally, one has to note that as in any functional neuroimaging study, the results presented in the present paper are of a correlational nature and, therefore cannot eliminate the possibility that the identified network is (partly) a result of other correlated factors, such as hearing loss or comorbid psychiatric problems. Future studies should extend the outcome by using TMS or TDCS to ground causal conclusions.

## Conclusion

Results of the present whole-cortex, directed functional connectivity analysis of the intrinsic networks of tinnitus patients and controls, indicated that the group of tinnitus patients showed increased connectivity between dorsal prefrontal and left medial temporal areas. Thereby, the results highlight the role of dmPFC, a region that is part of the dorsal attention network (DAN), as well as implicated to the regulation of emotional processing^38^, in modulating the left para-hippocampal area. Additionally, this whole cortex analysis showed an increased role of the left inferior parietal cortex, which modulated the activity of the right superior temporal gyrus, bilateral middle temporal and left visual association areas providing new hypotheses for the role of these areas within the context of current tinnitus models. Overall, these maladaptive alterations of the structure of the intrinsic cortical network show increased connectivity, but decreased efficiency and decreased processing economy in the resting state network of tinnitus patients. These alterations correlate with tinnitus-related distress and the Tinnitus Handicap Inventory.

## Methods

### Subjects

The sample of the present study consisted of 80 subjects, 40 chronic tinnitus patients and 40 controls. Tinnitus patients (mean age = 46.57; *SD* = 9.93; 23 males) suffered from tonal tinnitus with mean tinnitus duration of 9.79 years (*SD* = 7.3). Their mean tinnitus pitch was 5330 Hz (*SD* = 2245.2) and 26 of them suffered from bilateral tinnitus, while 9 suffered from unilateral left and 5 from unilateral right tinnitus. Alongside, tinnitus patients did not exhibit hearing loss above 70 dB HL in the frequency ranges of one half octave above and below the tinnitus frequency. Healthy controls had a mean age of 39.52 (*SD* = 12.05), while 24 of them were female. Controls were also tested via an audiometric procedure, indicating that they did not experience hearing loss greater than 20 dB HL in any frequency. Age and gender of the two groups did not differ significantly (*p* > 0.05). The study protocol was approved by the Ethics committee of the Department of Psychology of the University of Münster (AZ: 2011–109-f-S) and was conducted according to the Declaration of Helsinki. Each subject signed informed consent prior to the participation. Recruitment was conducted via advertisements in local newspapers, flyers in public places, doctor’s offices (ENT) and the webpage of the Institute for Biomagnetism and Biosignalanalysis of the University of Münster.

### MEG recordings - instrumentation

Resting state magnetic fields of the whole brain were recorded for 5 minutes in a magnetically shielded and acoustically quiet room via a 275 channel whole-head MEG system (OMEGA, CTF Systems Inc, Port Coquitlam, Canada). Data were continuously acquired using a sampling rate of 600 Hz. Subjects were seated upright, and their head position was comfortably stabilized inside the MEG dewar using pads and it was constantly monitored. Subjects looked at a black screen including solely a fixation cross, during data acquisition, having their eyes open and fixated at the cross presented on the screen, while they were instructed ‘to relax doing nothing’. The screen [52 × 40 cm (W × H)] was located at a distance of 90 cm from the subjects’ nasion. The subject’s alertness and compliance were verified by video monitoring. The recordings were performed prior to further MEG acquisitions used for other studies^55,56^. It has to be noted here, that the data included in the present study were subjected to a different analysis in^57^, which focused on activation differences between the groups and did not include any estimation of connectivity. Tinnitus patients also underwent a physical examination of the ear by an ENT physician, and completed behavioral measures of Tinnitus Handicap Inventory (THI)^58^ and as well as Visual Analog Scales measuring perceived tinnitus loudness, distress and handicap. The tinnitus frequency was estimated by a recursive two-interval forced choice procedure as described in^57^. All participants underwent a determination of hearing thresholds using a clinical audiometer (Type Madsen Astera, Denmark) that is able to operate in an extended frequency range up to 16 kHz.

### MEG data analysis

#### Pre-processing and Source activity estimation

Pre-processing of the MEG data was performed using the Brain Electrical Source Analysis software (BESA research, version 6, Megis Software, Heidelberg, Germany). The raw MEG signal was initially decomposed into independent components using the extended ICA algorithm^59^, in order to identify and exclude from further analysis the strongest components corresponding to ocular, cardiac, and muscle artifacts. The dimensionality of the data was reduced into 40 components using Principal Component Analysis prior to the use of the ICA. The continuous data stream was then filtered using a Butterworth high pass forward filter of 0.5 Hz and a notch filter at 50 Hz (power line frequency). In the following, the data were segmented into 8 epochs of 8 seconds each. The epochs were chosen pseudo-randomly from the complete recording, excluding the first minute (hence, they were chosen from the 2^nd^, 3^rd^, 4^th^ and 5^th^ minute of the recording), with no overlap and were further filtered using a Butterworth low pass zero-phase of 35 Hz.

Current density reconstructions (CDR) were calculated on the neural responses of each subject and for each complete epoch using the LORETA method^28^ as provided by BESA. LORETA has the advantage of not needing an *a priori* definition of the number of activated sources and may therefore provide a whole cortex reconstruction indicating multiple sources. A template lead-field based on an average Finite Element Model, a realistic approximation to the averaged head in Talairach space, as provided by the BESA software was used for all subjects. The complete frequency bandwidth of 0.5 to 35 Hz was used for the LORETA calculation. The voxel size for the inverse solution was defined as 10 mm and the CDR was calculated for each sample point of each epoch. The CDRs were exported as four-dimensional (4D) nifti images. These images were then processed with a mask that included only the gray matter, excluding the ventricles, the white matter, the brainstem and the cerebellum, in order to limit the source space. This process resulted in a source space of 863 voxels, while a node of the network was appointed to each of the voxels, covering the complete cortex.

#### Connectivity analysis – Statistical procedures

The activity of each voxel was exported as time series in order for the connectivity matrices to be calculated. The Phase transfer entropy (PTE)^29^ function for MATLAB (The MathWorks Inc., Natick, MA, US) as implemented by^60^ was used for the estimation of the 863 × 863 adjacency matrix from the voxel time series of each epoch. PTE is a directed connectivity measure that estimates phase-based information flow (and its direction) based on the transfer entropy between the time series^61^. The identification of the direction of the information flow is based on a cause – effect relationship indicating which region’s signal predicts the signal of (which) other regions and hence, modulates it. This is achieved by the estimation of non-symmetric connectivity matrices which provide the reason for directional connectivity inferences to emerge from the data. The main advantage of PTE is that, it is based on nonlinear probability distributions and therefore, it detects higher order relations within the signal’s phase information flow, while at the same time it is immune to source leakage^29^. Hence, its result is not dependent on any specific model of the data. In addition, the estimation of PTE is performed independently for every edge of the network, minimizing the dependency of the extent of the network tested (number of nodes included) to the amount of the data^29^. The delay of the transfer entropy (i.e. the time delay from the past of both the source as well as target to the next value of the target) was left undefined as in this way the algorithm calculates it as the average time between sign flips of the phase (averaged over all channels). Hilbert transformation is used to determine the phases of the signals. The number of time steps of the past of the target (i.e. embedding length k), was set to 1. This setting does not eliminate information from the past of the target being included as transferred from the source, a practice that has been recently criticized^62^ as it may lead to inflated PTE values. The bin size for the histograms of phase occurrences was determined following the approach proposed by^63^, resulting in, at least, 49 bins for each signal and hence 49 × 49 × 49 = 117649 overall bins. A sanity check of the data was performed by computing the PTE for two randomly chosen time series extracted from one epoch of one subject; the one of the two time series was then reversed and PTE was recomputed, which resulted in the flipping of the direction of the estimated connectivity. The adjacency matrices of the different epochs of each subject were then averaged (after the calculation of PTE) resulting in one connectivity matrix per subject. The connectivity matrix of each subject was then transformed into standardized scores (z-values).

**Figure 4:**
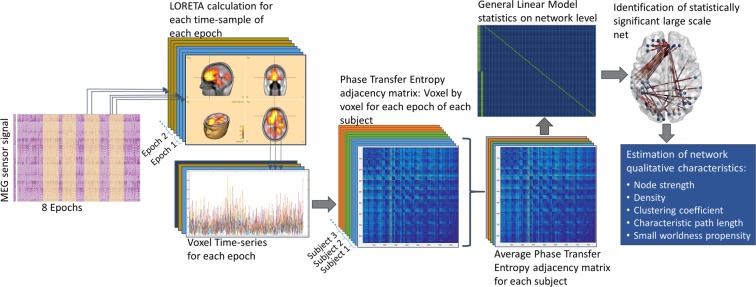
Illustration of the data analysis procedure. The MEG signal was segmented in eight equal and pseudorandom segments. A LORETA solution was applied for each time-sample of each segment estimating the corresponding source activity. The voxel time-series for each epoch were extracted and their directed functional connectivity was estimated via pTE. The average adjacency matrix based on pTE from all segments was calculated for each individual, and subjected to a statistical comparison, which identified significant networks and differences between the groups. The global qualitative characteristics of the network were estimated on the basis of the significant networks.

The Network Based Statistic (NBS)^64^ toolbox was used to identify statistically significant connections in the networks using general linear model. Specifically, the resting state network of each subject group (tinnitus patients and controls) was calculated comparing, via a t-test, the z-transformed connectivity matrix of each subject to a permuted set of data, generated by a procedure that randomly flips the sign of each data point of the adjacency matrix for each permutation^64^. Briefly, in this procedure a pre-multiplication of the full design matrix by a permutation matrix shuffles the order of the data, while multiplication by a sign flipping matrix additionally changes the sign of a random subset of data points^65^. Permutation and sign flipping are performed at the same time to construct the empirical distribution. Permutation tests permit the use of the hypothesis testing framework followed by the parametric procedures without the need to evaluate the validity of the assumptions needed for the corresponding parametric tests^65^. The interpretation of the outcome is the rejection of the null hypothesis of equality of means of the two groups on an independent, link-by-link basis^64^. Hence, it compares the sets of original z-transformed link weights for each connected pair. An independent samples t-test was also used to compare the resting state network of the two groups, in order to identify significant differences between the resting state network of tinnitus patients and controls. The significance level of the analyses, for both, was set to p < 0.001 corrected for multiple comparisons via False Discovery Rate (FDR) correction using a set of 5000 permutations. The visualization of the significant networks as weighted graphs was performed using BrainNet Viewer^66^. The complete analysis procedure is outlined in Fig. 4. The exact same analysis pipeline was applied to a set of surrogate data generated for each epoch’s timeseries and for each subject, using the corresponding algorithm included in the HERMES toolbox^67^ for MATLAB.

Lastly, after the identification of the statistically significant networks of each group, the graph characteristics of node strength, network density, efficiency, characteristic path length and small worldness propensity were estimated. The above mentioned characteristics were estimated using the Brain Connectivity Toolbox^68^, apart from small worldness propensity, which was estimated using the network community toolbox^47^. Node strength sums the weights (i.e., t values in the present analysis) of links connected to the node, identifying the nodes that show greater connectivity differences. The fraction of present connections to possible connections, is depicted by network density, indicating how many of the available cortical areas contribute to the network. Node strength and network density were calculated as qualitative descriptors of the statistically significant networks. The global efficiency is the average inverse shortest path length in the network, reflecting the level of global integration in the network. This index depicts the average number of nodes through which the information flows to reach a distant point of the network. The characteristic path length is the average shortest path length in the network, while small worldness propensity estimates whether the network has high local clustering but short average path length, taking into account the weights of the edges^47^. Small worldness describes topologically a network in being both economic as well as efficient in the synchronizability and information flow^30^. A non-parametric Mann-Whitney U test was performed to compare the graph characteristics of small world propensity, characteristic path length and efficiency of the networks between the two groups, as the results violated normality. Density was estimated on the basis of the statistically significant results.

Lastly, Pearson’s correlation coefficient between the graph measures of small worldness propensity, characteristic path length and efficiency with THI, loudness, distress and hearing loss were estimated using IBM SPSS Statistics for Windows, Version 24.0.

## Data Availability

The datasets generated during and/or analyzed during the current study are available from the corresponding author on reasonable request.
